# Serum proteomic profile of wild stump-tailed macaques (*Macaca arctoides*) infected with malaria parasites in Thailand

**DOI:** 10.1371/journal.pone.0293579

**Published:** 2023-11-01

**Authors:** Pakorn Ruengket, Sittiruk Roytrakul, Daraka Tongthainan, Kanokwan Taruyanon, Bencharong Sangkharak, Paviga Limudomporn, Mongkol Pongsuchart, Chanya Udom, Wirasak Fungfuang

**Affiliations:** 1 Genetic Engineering and Bioinformatics Program, Graduate School, Kasetsart University, Bangkok, Thailand; 2 Functional Ingredients and Food Innovation Research Group, National Center for Genetic Engineering and Biotechnology (BIOTEC), National Science and Technology Development Agency, Pathum Thani, Thailand; 3 Faculty of Veterinary Medicine, Rajamongala University of Technology Tawan-ok, Chonburi, Thailand; 4 Department of National Parks, Wildlife Conservation Division Protected Areas Regional Office, Wildlife and Plant Conservation, Ratchaburi, Thailand; 5 Department of National Parks, Wildlife Conservation Division, Wildlife and Plant Conservation, Bangkok, Thailand; 6 Faculty of Science, Department of Zoology, Kasetsart University, Bangkok, Thailand; Fundação Oswaldo Cruz Centro de Pesquisas René Rachou: Fundacao Oswaldo Cruz Instituto Rene Rachou, BRAZIL

## Abstract

The number of patients infected with simian malaria is gradually increasing in many countries of Southeast Asia and South America. The most important risk factor for a zoonotic spillover event of malarial infection is mostly influenced by the interaction between humans, monkeys, and vectors. In this study, we determine the protein expression profile of a wild stump-tailed macaque (*Macaca arctoides*) from a total of 32 blood samples collected from Prachuap Kiri Khan Province, Thailand. The malarial parasite was analyzed using nested polymerase chain reaction (PCR) assays by dividing the samples into three groups: non-infected, mono-infected, and multiple-infected. The identification and differential proteomic expression profiles were determined using liquid chromatography with tandem mass spectrometry (LC-MS/MS) and bioinformatics tools. A total of 9,532 proteins (total proteins) were identified with the filter-based selection methods analysis, and a subset of 440 proteins were found to be different between each group. Within these proteins, the GhostKOALA functional enrichment analysis indicated that 142 important proteins were associated with either of the organismal system (28.87%), genetic information processing (23.24%), environmental information processing (16.20%), metabolism (13.38%), cellular processes (11.97%), or causing human disease (6.34%). Additionally, using interaction network analysis, nine potential reporter proteins were identified. Here, we report the first study on the protein profiles differentially expressed in the serum of wild stump-tailed macaques between non, mono, and multiple malarial infected living in a natural transmission environment. Our findings demonstrate that differentially expressed proteins implicated in host defense through lipid metabolism, involved with TGF pathway were suppressed, while those with the apoptosis pathway, such as cytokines and proinflammation signals were increased. Including the parasite’s response via induced hemolysis and disruption of myeloid cells. A greater understanding of the fundamental processes involved in a malarial infection and host response can be crucial for developing diagnostic tools, medication development, and therapies to improve the health of those affected by the disease.

## Introduction

Malaria is an infectious disease that remains a global public health issue as it still causes mortality and morbidity in tropical and subtropical areas[1,2] In particular, the number of malaria cases in Southeast Asian region has considerably decreased by 78% from 23 million in 2000 to 5 million in 2020, with the case incidence rate having decreased by 83% [3]. However, the World Health Organization (WHO) has recently reported that approximately 241 million cases of malaria were registered in 2020, which was up from 227 million cases in 2019, with the disease being endemic in 85 countries (including the territory of French Guiana), with countries in the WHO African region accounting for most of this increase [3]. Because of the coronavirus disease 2019 (COVID-19) pandemic disrupting normal healthcare services, the incidence of malaria increased from 56 cases per 1000 persons in 2019 to 59 cases per 1000 persons in 2020 [3]. Moreover, the zoonotic spillover of malarial infections to humans is emerging as a new challenge to the goal of a complete eradication of malaria in Southeast Asia and South America [4,5]. Environmental changes, such as deforestation, changes in land use for agriculture, fragmented landscapes, and increased urbanization, are the main reasons behind increased interactions between humans and macaques [6], which are the primary carriers of malarial parasites. Furthermore, agricultural labor in forests, forest-edges, and other human activities, such as camping, hiking, and eco-tourism, have led to an increase in the likehood of contact with the *Anopheles* mosquitoes and the monkey populations [7].

Malaria is caused by a protozoan parasite of the genus *Plasmodium* and is transmitted by the females of the *Anopheles* Leucosphyrus groups[1,8]. More than 250 *Plasmodium* sp. parasites are have been isolated in several vertebrate hosts, including rodents, birds, reptiles, and mammals [9,10]. Recently, over 30 *Plasmodium* parasites have been reported in non-human primates, including Old, and New-world monkeys, apes, and gibbons [9,11]. Numerous non-human primate malarial infections have been reported as a result of natural and experimental transmission of parasites such as *P*. *knowlesi*, *P*. *cynomolgus*, and *P*. *inui* from the Old-world monkeys, and *P*. *simium* and *P*. *schwetzi* from the New-world monkeys and chimpanzees to the humans, respectively [12–15].

The first reported natural infection with *P*. *knowlesi* in humans was reported in the year 1965 in an individual serving in the American army [16]. However, the previous study reported a high number of humans infected with *P*. *knowlesi* in the Sarawak state of Malaysian Borneo [17]. Over the past decade, the cases of humans naturally infected by *P*. *knowlesi* have been reported in many Southeast Asia countries, including Singapore, Thailand, Laos, Vietnam, Indonesia, Philippines, Myanmar, Cambodia, and Brunei Darussalam [18–22]. Additionally, travelers returning from countries where the disease endemic, have also been reportedly infected with *P*. *knowlesi* [23,24]. In Thailand, the first case of natural *P*. *knowlesi* infection in humans was reported in 2004 [18], with the number of cases having increased considerably since then, as reported by the Thailand Malaria Elimination Program, which reported 122 cases in 2022 [25]. To date, natural *P*. *cynomolgi* infection in humans has been studied in the Tak, Ubon Ratchathani, Chantaburi, Yala, and Narathiwat provinces during the years 2007–2017 [26] and in the Yala Province in 2022 [27]. Additionally, apart from *P*. *knowlesi*, other simian parasites have also been identified in the naturally acquired human infections in Southeast Asia.

The first natural human infection with *P*. *cynomolgi* was reported in Peninsular Malaysia, in 2014 [28]. Subsequent infections with *P*. *cynomolgi* were reported in Cambodia, Malaysia, and Thailand [27,29–32]. Furthermore, infection with *P*. *cynomolgi* was reported in a Danish tourist visiting Thailand and Peninsular Malaysia [33]. The cases of humans infected with *P*. *inui*, *P*. *coatneyi*, *P*. *simiovale*, and *P*. *inui*-like parasites have also been reported in Malaysia [34,35]. The predominant natural hosts of *P*. *knowlesi*, *P*. *cynomolgi*, *and P*. *inui* are long-tailed *(M*. *fascicularis*), and Pig-tailed (*M*. *nemestrina*) [36,37] macaques, which are widespread in Southeast Asia. Other studies have also reported *P*. *knowlesi* infections in banded (*Presbytis melalophos*) and dusky (*Semnopithecus obscurus*) leaf monkeys of Peninsular Malaysia [38] and Thailand [39], respectively. Previously, our study reported that *M*. *arctoides* is a natural host for *P*. *knowlesi*, *P*. *inui*, *P*. *coatneyi*, and *P*. *fieldi* parasites [14]. *P*. *knowlesi* is the most prevalent *Plasmodium* parasite in the macaque population of Malaysia, whereas *P*. *inui* and *P*. *cynomolgi* are the most predominant parasites in other countries [40]. However, further research on malarial infection in wild macaques is needed to determine its potential risk to public health.

Serum is commonly used as a useful diagnostic and prognostic biomarker to test for the presence of several diseases and metabolites, as different types of proteins are released from various diseased tissue into the serum [41,42]. During the last decade, serum/plasma proteomics has extensively been used to detect protein markers of several diseases, including cancer and metabolic and infectious diseases [43]. Recently, this proteomics was used in researching malaria to provide information on the relevant parasite and host proteins to isolate various protein complexes involved with the cellular and pathogenic mechanisms, parasite proteomics, specific functions, vaccine development, and mechanism of drug action [44]. A previous study indicated that differentially expressed proteins modulate multiple functions such as acute phase response signaling, complementing and coagulation cascades, hemostasis and vitamin D metabolism during a *P*. *vivax* infection in humans [45]. Another study explained the alterations in protein expression in human serum induced by *P*. *vivax*, including Haptoglobin, Apolipoprotein E, Apolipoprotein a1, C-reactive protein, Titin and Haptoglobin for different levels of malarial parasitemia. Given the close evolutionary relationship between humans and macaques, they share the basic elements of the immune system [41]. However, no serum proteomics analysis has been performed in wild macaques infected with the *Plasmodium* parasite. This begs a need for a proteomics analysis to delve into the pathogenic mechanisms of zoonotic malarial infection and to further understand the host response to such parasites.

In this study, we investigated the host serum proteomic profile of wild *M*. *arctoides* naturally infected with malarial parasites using a proteomics approach and bioinformatics methods, including supervised classification prediction and unsupervised clustering analysis. Our study isolated the differentially expressed protein profile between the non-infected, mono-infected, and multiple-infected groups. These findings provide insights into a host’s response to the infection and will improve our knowledge related to the pathophysiology of natural malarial infection in nonhuman primates between the non, mono, and multiple-infection groups. This knowledge would be key to treatment decisions, fast healing, and effective patient management and would also help in preventing severe malaria (multiple-infections) from crossing over to humans.

## Materials and methods

### Ethics statement and blood sample collection

All the animal care and handling procedures were approved by the Institutional Animal Care and Use Committee of the Kasetsart University Research and Development Institute, Kasetsart University, Thailand (ID: ACKU59-SCI-011). The experimental protocols for research in the conservation area were approved by the Department of National Parks, Wildlife and Plant Conservation, Thailand (Permit Number: 0909.204/14187).

Blood samples were collected from 32 wild stump-tailed macaques (*M*. *arctoides*) at the Pa La U waterfall, Huahin district, Prachuap Kiri Khan Province, Thailand in December 2018. All macaques were trapped and anesthetized using an intramuscular injection of Tiletamine and Zolazepam (Zoletil^®^, Virbac, Hamilton, New Zealand) at a dosage of 2–5 mg/kg and Xylazine hydrochloride at a dosage of 0.5–2 mg/kg. A blood sample of not more than 3 ml was drawn from the femoral vein of each macaque into a tube containing ethylenediaminetetraacetic acid (EDTA). In the laboratory, 2200 g of blood sample was centrifuged for 20 min at 4°C and the serum thus obtained was stored at -80°C until further processing. All macaques were released after they recovered from the anesthesia at the collection site. In addition, 40–50 μl of blood samples were transferred to Whatman 3MM filter paper as a storage medium for dried blood spots and kept at room temperature for the DNA extraction and molecular analysis.

### *Plasmodium* identification by nested polymerase chain reaction (PCR) assay

The dried blood spots were sent to the Malaria Research Centre, University of Malaysia, Sarawak, Malaysia, to identify the *Plasmodium* species through molecular analysis. The DNA was extracted from the dried blood spots using InstaGene (Bio-Rad Laboratories, USA), as described previously [46]. The DNA samples were initially analyzed through a nested polymerase chain reaction (PCR) with genus-specific primers for detecting *Plasmodium*, as described previously [47]. The *Plasmodium*-positive samples were then analyzed through a nested PCR with species-specific primers for *P*. *knowlesi*, *P*. *coatney*, *P*. *cynomolgi*, *P*. *inui*, and *P*. *fieldi* parasites, as described previously [48].

### Serum preparation

The protein content of each serum sample was measured according to the Lowry protein assay protocol using bovine serum albumin as a protein standard [49]. Disulfide bonds were reduced by adding 5 mM dithiothreitol in 10 mM ammonium bicarbonate to 5 μg serum and incubating at 60°C for 1 h, followed by sulfhydryl group alkylation with 15 mM iodoacetamide in 10 mM ammonium bicarbonate at room temperature for 45 min in the dark. Subsequently, the carbamidomethylated protein samples were digested with sequencing-grade trypsin at a ratio of 1:20 and incubated at 37°C overnight. The tryptic peptides were dried using a speed vacuum concentrator, and the pellet was resuspended in 0.1% formic acid for nano liquid chromatography-tandem mass spectrometry (Nano LC-MS/MS) analysis.

### Nano LC-MS/MS analysis

Tryptic peptide samples were prepared for injection into an Ultimate3000 Nano/Capillary LC system (Thermo Scientific, UK) coupled to a HCTUltra LC-MS system (Bruker Daltonics Ltd; Hamburg, Germany) equipped with a nano-captive spray ion source. Briefly, 5 μg of peptide digests were enriched on a μ-Precolumn with a 300 μm inner diameter (ID) X 5 mm C18 Pepmap 100, 5 μm, 100 A (Thermo Scientific, UK), separated on a 75 μm ID x 15 cm and packed with Acclaim PepMap RSLC C18, 2 μm, 100 Å, nanoViper (Thermo Scientific, UK). The C18 column was enclosed in a column oven set to 60°C. Solvents A and B containing 0.1% formic acid in water and 0.1% formic acid in 80% acetonitrile, respectively, were supplied to the analytical column. The peptides were eluted by a gradient of 5–55% of solvent B at a constant flow rate of 0.30 μl/min for 30 min. Electrospray ionization was performed at a voltage of 1.6 kV using CaptiveSpray. Nitrogen was used as a drying gas (flow rate about 50 l/h). The collision-induced-dissociation (CID) product ion mass spectra were recorded using nitrogen as the collision gas. MS and MS/MS spectra were acquired in a positive-ion mode at 2 Hz over a range of 150–2200 (m/z). The collision energy was adjusted to 10 eV as a function of the m/z value and each sample was analyzed through LC-MS in triplicates.

### Protein identification and quantification

DeCyder MS differential analysis software was used to quantify the peptides in the LC-MS/MS data and a Mascot search engine was used to correlate the MS/MS spectral data with the UNIPROT protein database to determine the *Macaca* genus [50,51]. The following Mascot’s standard settings were used: a maximum of three miss tryptic cleavages, a fragment peptide mass tolerance of 1.2 Da, an MS/MS tolerance of 0.6 Da, trypsin as the digesting enzyme, cysteine carbamidomethylation (Cysteine CAM) as the fixed modification, methionine oxidation as the variable modification, and peptide charge states of 1+, 2+, and 3+. The protein expression levels (PELs) obtained from the MS/MS spectra were expressed as log2 values. The percentage of proteins unique to each sample was calculated as frequency of unique proteins divided by the total number of samples.

### Statistical analysis

The samples were divided into 16 non-infected, 9 mono-infected, and 7 multiple-infected groups. The PEL values were compared using the Kruskal-Wallis H test between the non-infected, mono-infected, and multiple-infected groups. The Mann-Whitney U test was used to compare differences between the non-infected and infected (mono-infected and multiple-infected) groups, and the same test was used to compare the differences between the mono-infected and multiple-infected groups. A confidence interval of 95% was used for both the Kruskal-Wallis H and Mann-Whitney U tests. The statistical analysis was performed on the IBM SPSS Statistical software version 22.0.0. After the statistical analysis, the proteins were divided into three subsets. Prior to the statistical analysis, correlates were constructed from all the proteins (totaling to 9,532) using the MS, MS/MS spectra, and UniProt proteome as the protein features. Double differentially expressed proteins (DDEPs; 440 in total) were determined using the significantly unique proteins isolated from two or more methods, while triple differentially expressed proteins (TDEPs; 31 in total) were determined from three methods. Protein upregulation and downregulation was then calculated from the differential PELs (median), and differential the percentages of proteins were identified in each group.

### Supervised and unsupervised machine learning analysis

Supervised machine-learning tools were used to classify data and includedfour classification algorithms: support vector machine (SVM), k-nearest neighbors (KNN), logistic regression (LR), and multinomial naive Bayes (MNB) classifier. A polynomial kernel of degree two was used for the SVM learning models with associated learning algorithms using a [52,53]. The k-NN is a non-parametric, supervised classification method and in this study, k-NN was run with number of neighbors (K) = 3, with Euclidean distance used as the distance metric [52,53]. LR uses a logistic function to model a binary output variable and the Broydene-Fletcher-Goldfarbe-Shanno (BFGS) algorithm has been used as an iterative method for solving unconstrained nonlinear optimization problems [52]. The MNB classifier is suitable for classification problems with discrete features, and for the present analysis, the parameter alpha was set to 0.1 in this model [54]. The accuracy score of the predicted classification for the samples was evaluated through a leave-one-out-cross-validation.

Unsupervised machine learning classifier was used to cluster the sample groups. The protein distribution and clustering was visualized as a scatter plot on the two principal axes obtained by the principal component analysis (PCA), with PEL data used as features. The 32 samples were analyzed by hierarchical clustering analysis (HCA) based on PELs, with the cluster samples divided into three main clusters, the non-infected, mono-infected, and multiple-infected groups.

### Data analysis

Gene orthologous and functional classification was performed using the EuKaryotic Orthologous Groups (KOGs) (https://www.hsls.pitt.edu/) and Kyoto Encyclopedia of Genes and Genomes or KEGG Orthologous (KO) (https://www.genome.jp/kegg/ko.html) databases. The KEGG Orthology And Links Annotation (GhostKOALA) (https://www.genome.jp/kegg/pathway.html) database was used to assign the putative protein functions as indicated by the K number of the protein sequence using HMMER under a cut-off E-value of 1E-0.5 and was used to described the protein pathway. Gene ontology was classified using the Protein Analysis Through Evolutionary Relationships (PANTHER) classification system (http://pantherdb.org/).

Protein-protein interaction networks were constructed using the STRING database version 11.5 (https://string-db.org/), with the following setting: a hight a minimum required interaction confidence score of 0.700 and no more than 10 interactors. The STITCH database version 5.0 (http://stitch.embl.de/cgi/) was used to construct the functional protein-chemical association networks and set at a medium confidence score of 0.400 for mapping proteins and chemicals associated with malaria. The STRING and STITCH databases were visualized using the Cytoscape software version 3.9.0. and were analyzed through Spearman’s rank coefficient of correlation.

## Results

### Simian malaria detection

**Fig 1:**
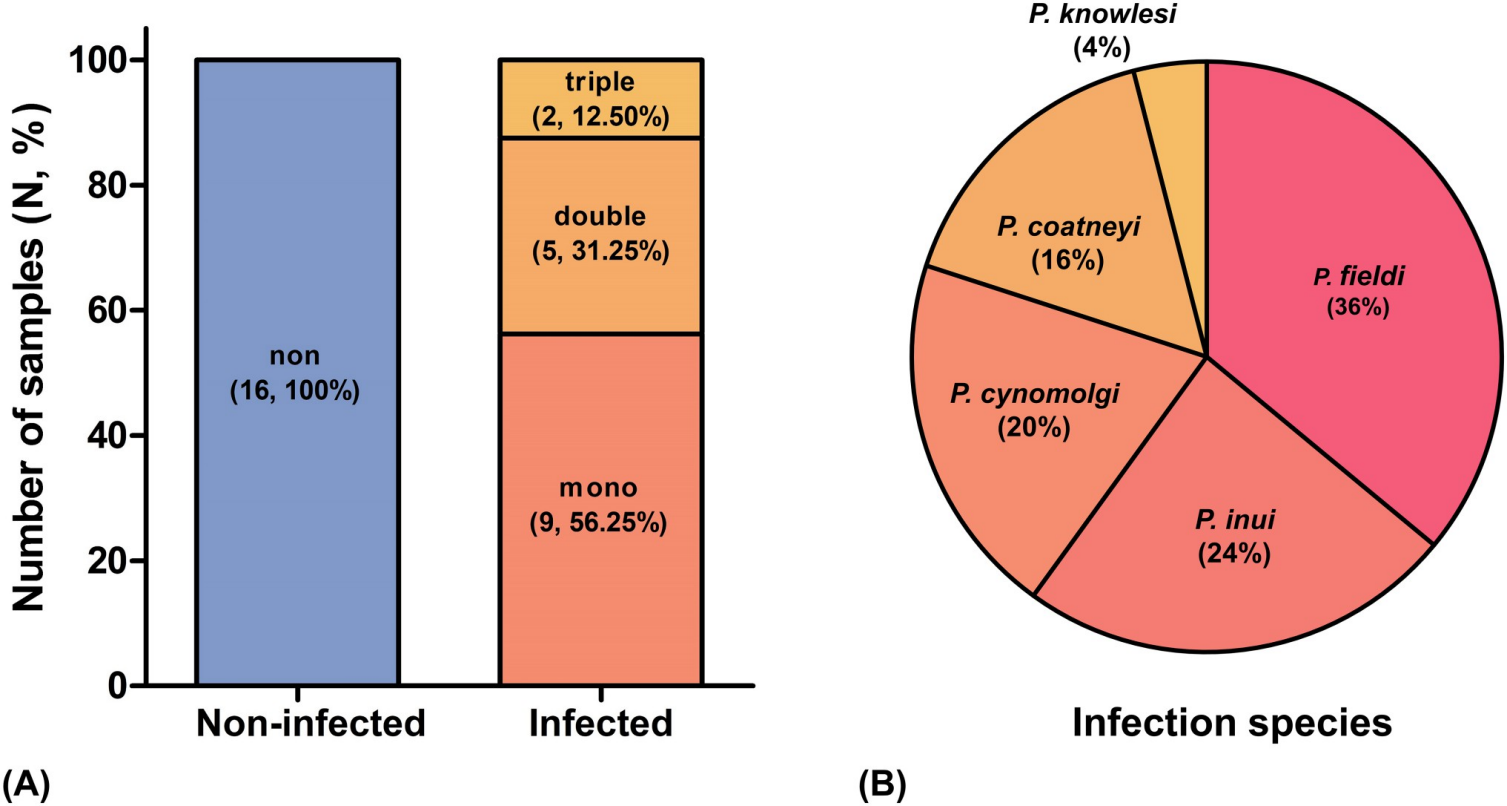
Simian malaria parasite identification and infection type. (A) The blue bar shows the percentage and numbers of samples in the non-infected group, whereas the red-yellow bar shows the percentage of each type of malaria infection. (B) Further elucidation of *Plasmodium* species as a percentage of the infected species.

In this study, a total of 32 wild stump-tailed macaques were screened for simian malarial parasites using nested PCR assays, 50% of which were positive for *Plasmodium* spp. Five species of *Plasmodium* were detected, namely *P*. *knowlesi*, *P*. *inui*, *P*. *fieldi*, *P*. *cynomolgi*, and *P*. *coatneyi* (Fig 1A). *P*. *fieldi* was the most prevalent species (36%) followed by *P*. *inui* (24%), *P*. *cynomolgi* (20%), *P*. *coatneyi* (16%), and *P*. *knowlesi* (4%) (Fig 1B). The frequency of mono, double, and triple infections was 56.25, 31.25, and 12.50%, respectively. Subsequently, the samples were divided into three groups; non-infected (50%), mono-infected (28.12%), and multiple-infected (21.88%), for a further serum proteomics analysis.

### Filtration of the proteomic profiling

**Fig 2:**
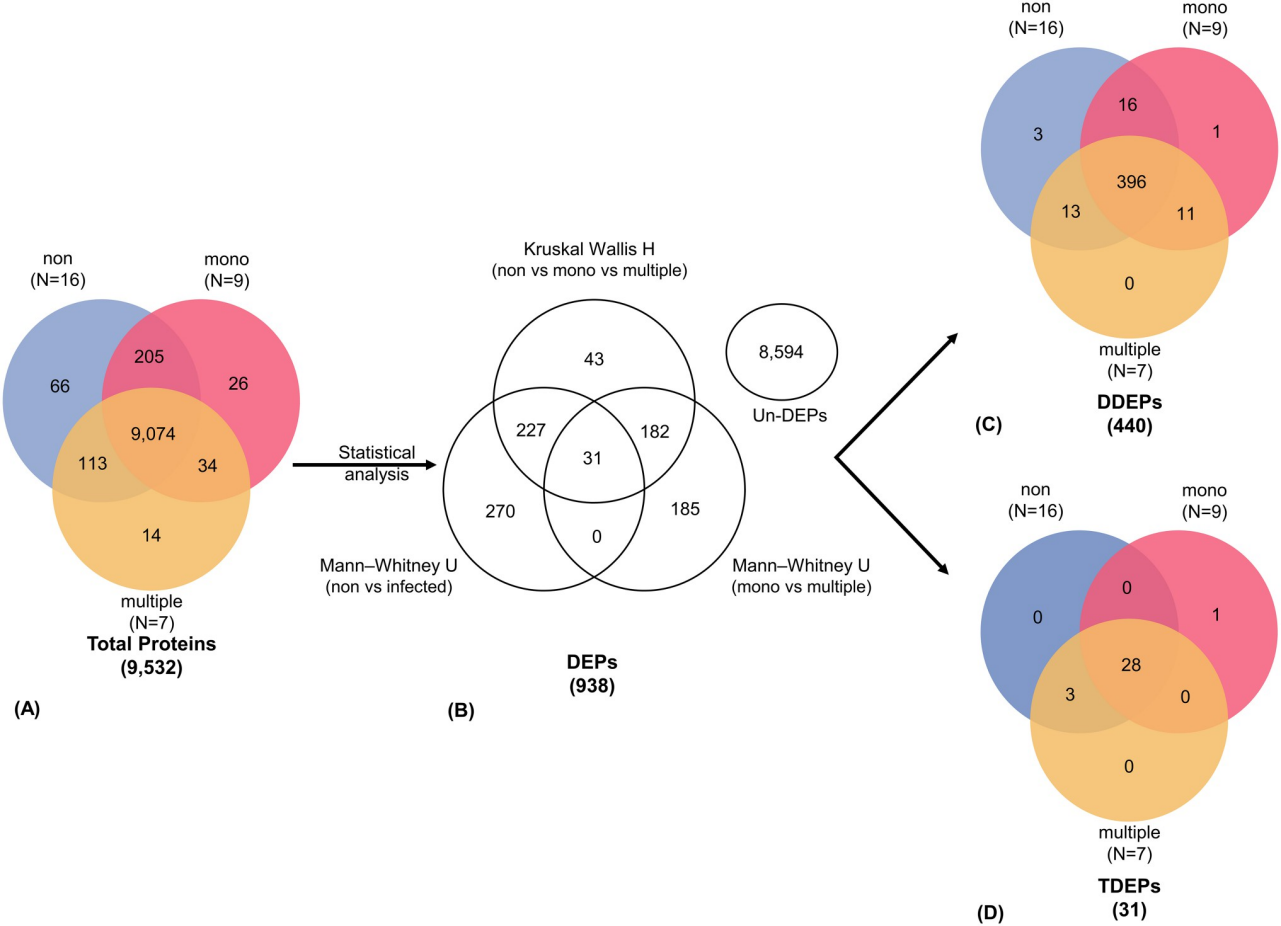
Descriptive data and filtration methods. (A) Venn diagram showing the protein expression in three groups. (B) The three methods of protein filtration methods resulted in differentially and un-differentially expressed proteins as indicated by statistical analysis. The Venn diagram shows unique proteins after filtration methods, (C) double differentially expressed protein subsets, and (D) triple differentially expressed protein subsets in each group.

A total of 9,532 proteins were identified by proteomic profiling. The Venn diagram shows the number of uniquely expressed proteins among the three groups (Fig 2A). Out of these, 66, 26, and 14 proteins were unique to the non-infected, mono-infected and multiple-infected groups, respectively. The expressed proteins common between two groups included 205 proteins in non-infected and mono-infected groups, 113 proteins in non-infected and multiple-infected groups, and 34 proteins in mono-infected and multiple-infected groups. Subsequently, the proteins were filtered using methods of statistical analysis (Fig 2B). A total of 473 proteins were different as indicated by the Kruskal-Walis H test, with 398 proteins being different in the non-infected and multiple-infected groups, while 528 proteins were different in mono-infected and multiple-infected, both indicated by Mann-Whitney U test. In addition, two protein subsets were constructed using the statistical analysis and included 440 DDEPs (Fig 2C), and 31 TDEPs (Fig 2D).

### Supervised machine learning classification for selected protein subsets

The supervised machine learning classification was used to identify protein subsets (total proteins, DDEPs, and TDEPs) to increase the prediction accuracy. The highest classification accuracy was obtained while identifying DDEPs. The classification accuracy of the four algorithms was estimated at 96.88% (1/32 samples incorrectly classified) (Table 1). This result indicated that the use of DDEPs as a classification feature yielded the best results. Although the three filtration statistical analyses methods of TDEPs gave a relatively high predictive accuracy, it was still lower than that of DDEPs.

**Table 1:** Supervised machine learning classification algorithm for select protein subsets.

Protein Subsets	Total Proteins	^a^DDEPs	^b^TDEPs
Number of features	9,532 Proteins	440 Proteins	31 Proteins
Classification algorithm	Accuracy	Accuracy	Accuracy
Support vector machine	50.00%	96.88%*	90.62%
K-nearest neighbors	53.12%	96.88%*	87.50%
Logistic regression	50.00%	96.68%*	90.62%
Multinomial Naive Bayes	46.88%	96.88%*	93.75%

^a^ DDEPs = double differentially expressed proteins.

^b^ TDEPs = triple differentially expressed proteins.

*Highest accuracy scores.

### Proteomic enrichment and profiling

**Fig 3:**
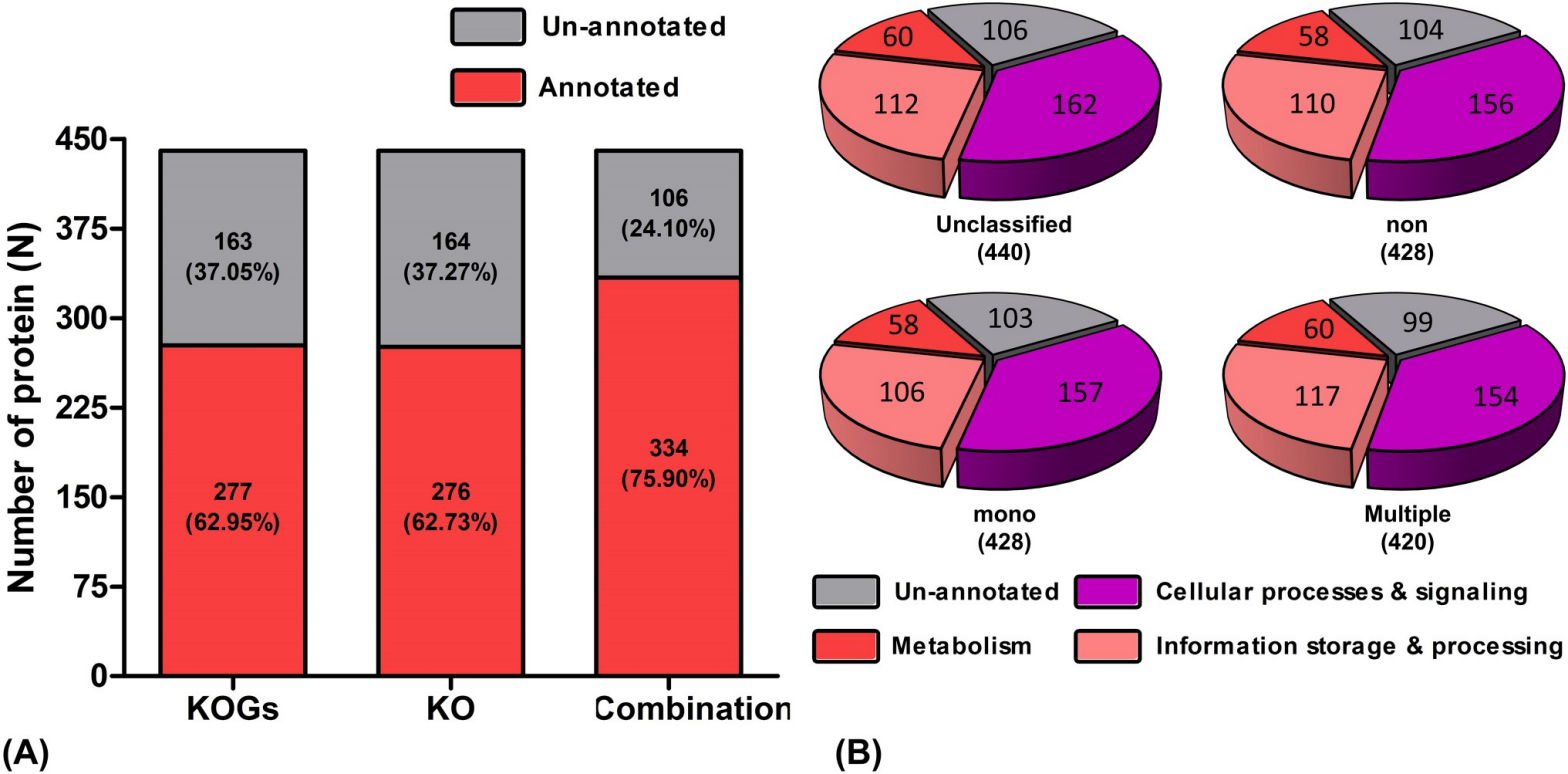
Gene orthologous based on the 440 DDEPs. (A) Bar chart showing the percentage and numbers of gene orthologous annotated proteins as obtained by KO and KOGs databases and combination databases. (B) The number of proteins identified in the unclassified, non-infected, mono-infected, and multiple-infected groups.

A total of 440 DDEPs were classified and catergorized using KOGs and KO databases. A total of 277 (62.95%) and 276 (62.73%) proteins were classified by KOGs and KO, respectively. A combination of the two database increased the annotation up to 334 proteins (75.90%) (Fig 3A). Subsequently, these DDEPs were classified into three categories according to their function in cell processes and signaling, information storage, and metabolism (Fig 3B).

**Fig 4:**
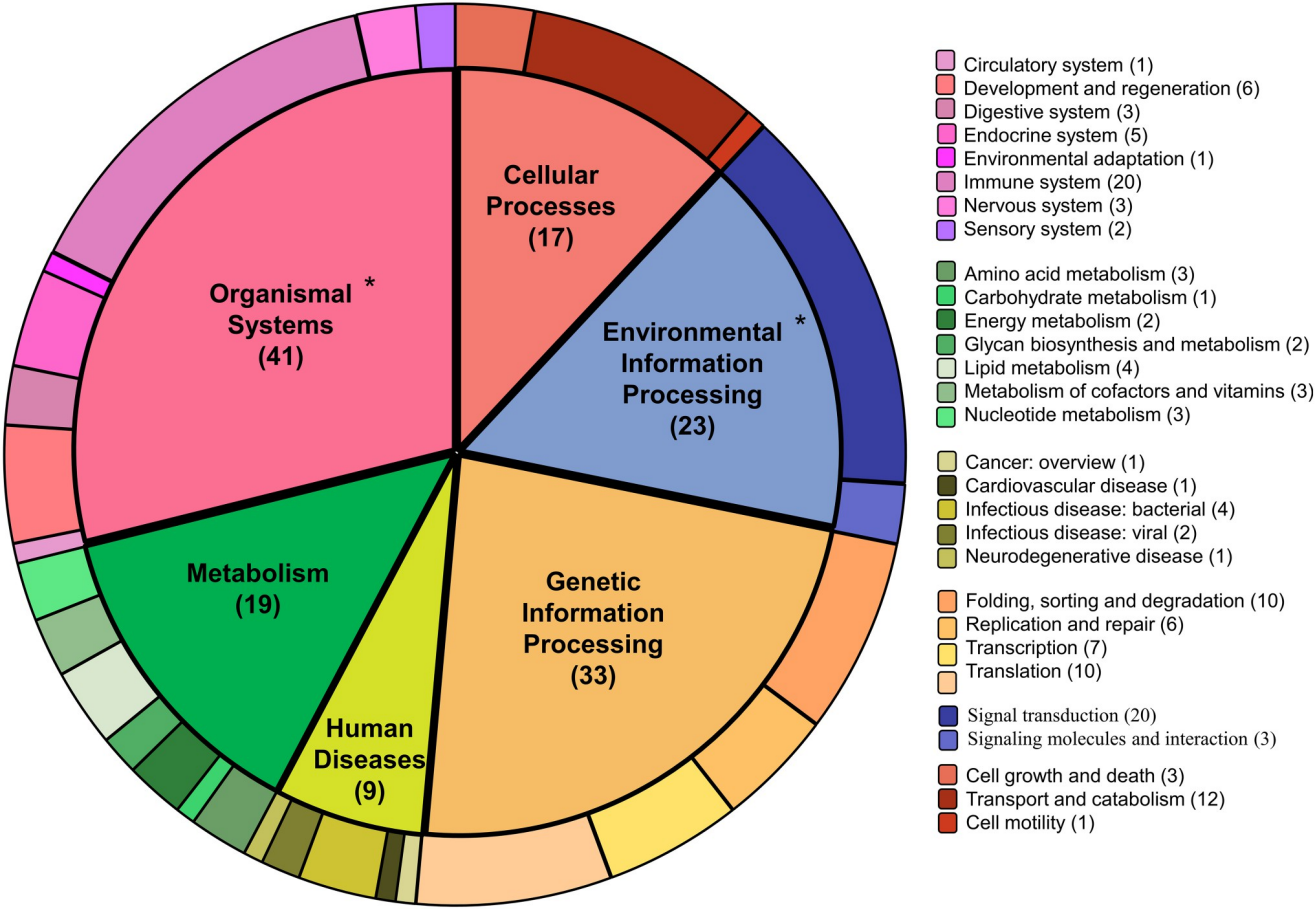
Described pathways based on the 142 important protein subsets. The pie chart shows 142 important protein subsets in pathways namely cellular processes, metabolisms, organismal systems, genetic information processing, environmental information processing, and human disease pathways.

To enrich the important proteins, the presence of the 334 annotation proteins were searched in the GhostKOALA databases (KEGG pathway) and 142 proteins were identified. These proteins, were classified into six pathway categories including organismal system (28.87%), genetic information processing (23.24%), environmental information processing (16.20%), metabolism (13.38%), cellular processes (11.97%), and causing human disease (6.34%) (Fig 4). However, we focused primarily on the organismal systems and environmental information processing pathways because of their relevance to host response to disease. The 142 protein subsets were re-analyzed through supervised machine learning classification and it was found that these protein subsets increased the accuracy scores of two algorithms, the SVM and MNB, as indicated by the absolutely prediction accuracy scores listed in Table 2.

**Table 2:** Supervised machine learning classification algorithm analysis for confirmed important protein subsets.

Protein Subsets	142 Protein Subsets
Number of features	142 Proteins
Classification algorithm	Accuracy
Support vector machine	100.00%*
K-nearest neighbors	96.88%
Logistic regression	100.00%*
Multinomial Naive Bayes	98.88%

*Increase accuracy scores.

**Fig 5:**
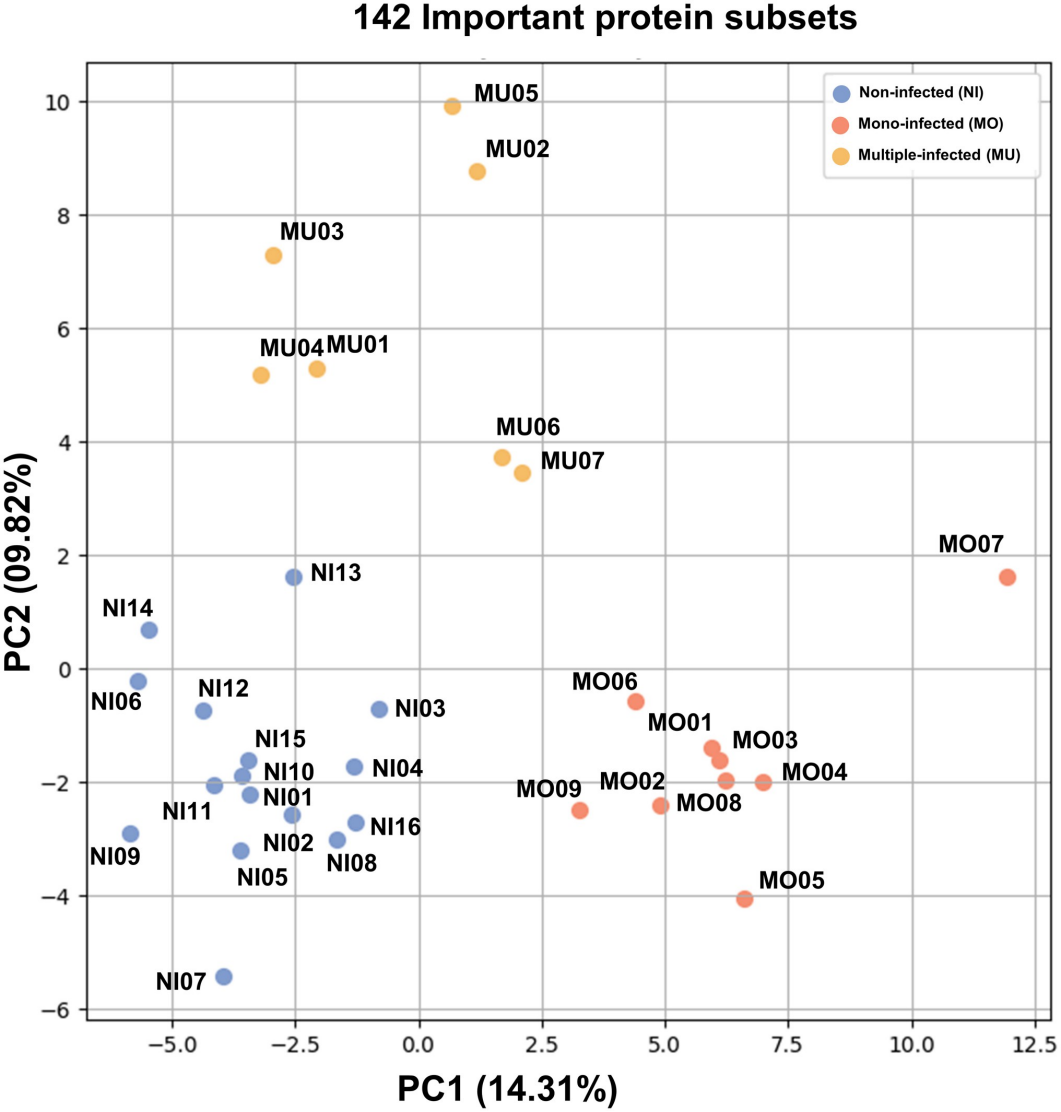
Unsupervised principal component analysis based on the 142 important protein subsets. The first and second components explained 14.59 and 9.36% of the total variance, respectively. Blue, red, and yellow dots represent the 32 samples in the non-infected, mono-infected, and multiple-infected groups, respectively.

**Fig 6:**
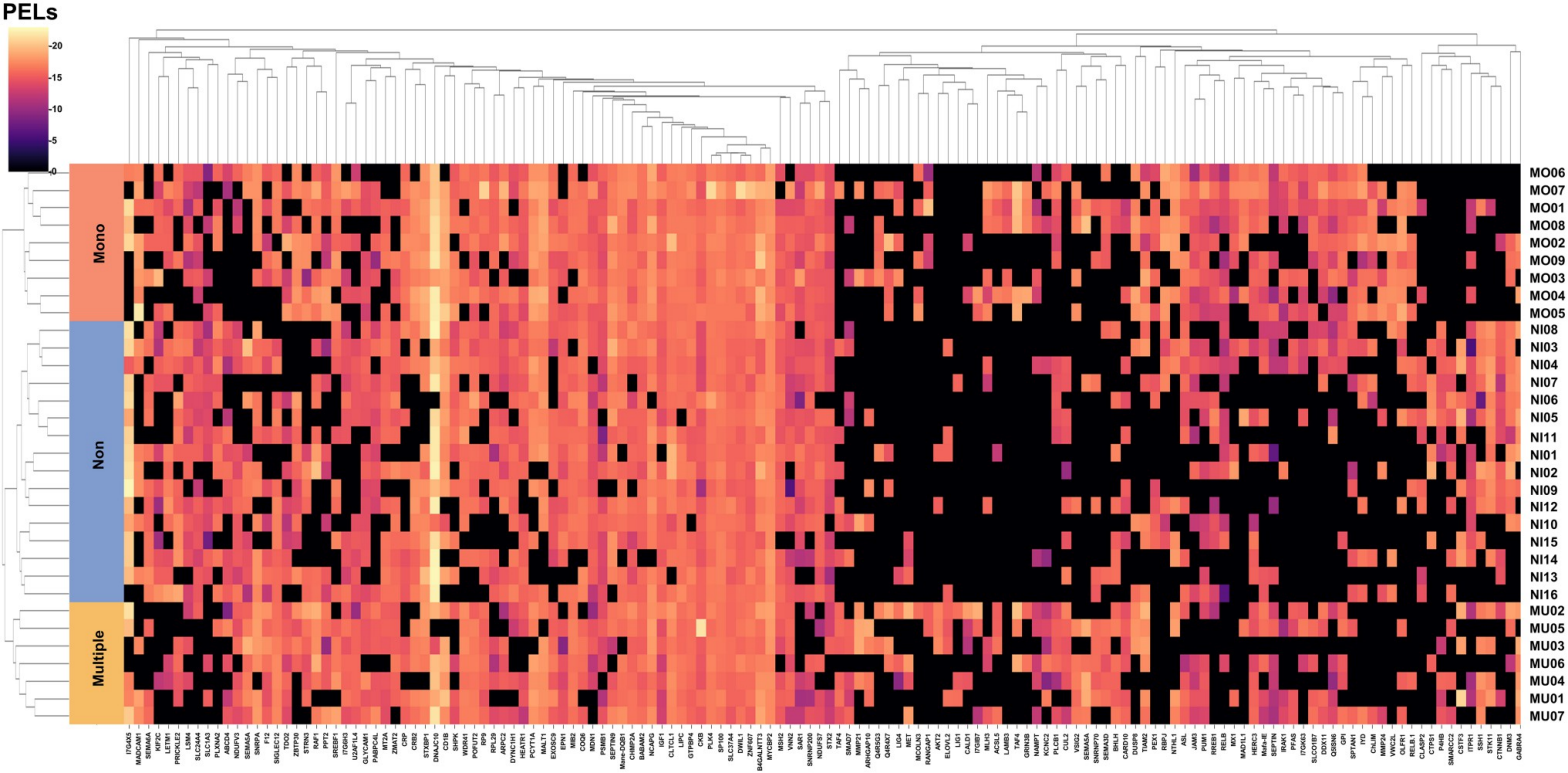
Unsupervised hierarchical clustering heatmap analysis based on the 142 important protein subsets. Blue, red, and yellow bands in the vertical rows represent samples in the non-infected, mono-infected, and multiple-infected groups, respectively. The horizontal columns indicate the 142 protein names. The color of the heatmap shows the scale of PELs with low-to-high expression in 32 samples and 142 protein features.

Furthermore, an unsupervised PCA of the 142 most important protein subsets was calculated and is summarized as a scatterplot (Fig 5). Together, the first two principle components were able to explain 24.13% of the variance in the data, with the contributions of the first and the second principle components being 14.31 and 9.82%, respectively. Fig 6 shows the heatmap obtained from the unsupervised hierarchical clustering analysis, which separated the proteins into three main clusters. Through the hierarchical clustering analysis, a clear separation was obtained between the non-infected, mono-infected, and multiple-infected groups and the cluster of protein expression levels.

### Identification of potential reporter proteins

**Fig 7:**
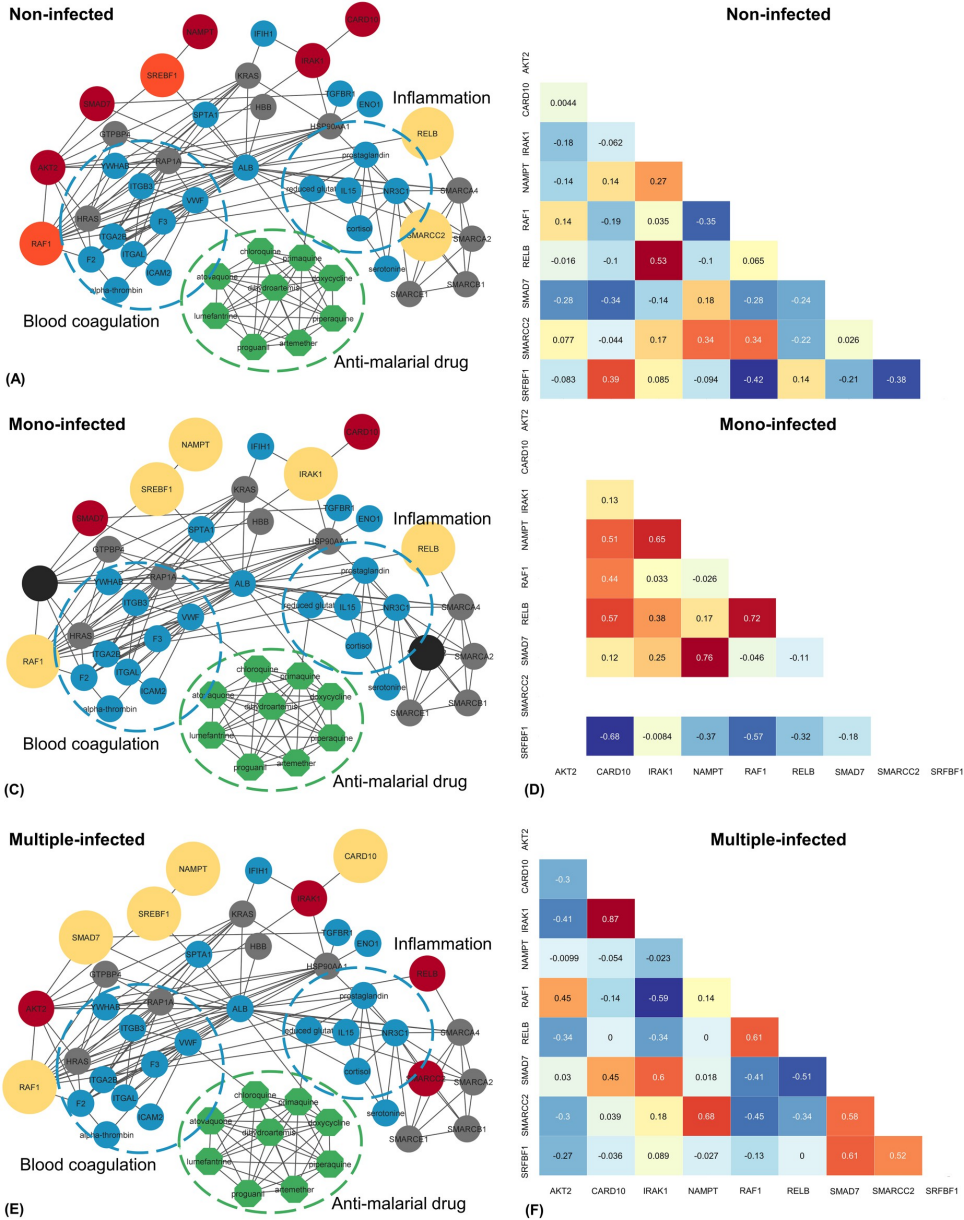
Construction of protein interaction networks. The combination of nine potential proteins between the protein-protein and protein-chemical interaction networks (A) in the non-infected, (C) the mono-infected, (E) and the multiple-infected. The Spearman’s rank correlation coefficient of nine potential proteins associated immune system and blood coagulation when the non-infected as feature proteins (B), mono-infected (D), and multiple-infected (F).

Amog the 142 important protein subsets, the protein-protein and proteins-chemical interaction networks were analysed by focusing on 23 proteins involved with the environmental information processing pathway and 41 proteins involved with the organismal systems pathway, which consider the host response to malaria, inflammation, and blood coagulation. Nine potential reporter proteins were identified in the interaction networks and correlated with each group (Fig 7). Interestingly, we identified three proteins that were up-regulated in the infected macaques (mono and multiple infections), namely the Sterol regulatory element binding transcription factor 1 (SREBF1), non-specific serine/threonine protein kinase (RAF1), and Nicotinamide phosphoribosyltransferase or NAmPRTase (NAMPT). The only protein found up-regulated in the mono-infected group was Interleukin 1 receptor associated kinase 1 (IRAK1). Caspase recruitment domain family member 10 (CARD10) and Mothers against decapentaplegic homolog (SMAD7) were uniquely up-regulated in the multiple infection group. Nevertheless, the results indicate that the SWI/SNF complex subunit SMARCC2 isoform c (SMARCC2) was down-regulated in the infected group. In addition, RELB proto-oncogene, NF-kB subunit (RELB) was up-regulated in the non-infected and mono-infected groups, while AKT serine/threonine kinase 2 (AKT2) was up-regulated in the non-infected and multiple-infected groups (Table 3).

**Table 3:** Nine potential reporter proteins.

Initial	IDs	Protein names	PELs^a^			% Protein identified ^b^		
			NI	MO	MU	NI	MO	MU
SREBF1	F7E4A8	Sterol regulatory element binding transcription factor 1	7.31	16.98*	16.53*	50.00	88.89*	71.43*
RAF1	A0A2K6DFP8	Non-specific serine/threonine protein kinase	7.18	16.28*	17.34*	50.00	88.89*	85.71*
NAMPT	A0A2K5ULM8	Nicotinamide phosphoribosyltransferase	0.00	11.65*	12.59*	12.50	55.56*	71.43*
IRAK1	A0A2K6B010	Interleukin 1 receptor-associated kinase 1	0.00	13.56*	0.00	18.75	77.78*	42.86
CARD10	A0A2K6CAT5	Caspase recruitment domain family member 10	0.00	0.00	14.67*	37.50	33.33	85.71*
SMAD7	I0FRQ6	Mothers against decapentaplegic homolog	0.00	0.00	13.88*	37.50	22.22	71.43*
SMARCC2	I2CXI3	SWI/SNF complex subunit SMARCC2	14.03*	0.00	0.00	56.25*	0.00	42.86
RELB	A0A2K5V1L0	RHD domain-containing protein	12.07*	13.08*	0.00	68.75*	88.89*	14.29
AKT2	A0A1D5RH80	AKT serine/threonine kinase 2	0.00	0.00	0.00	12.50*	0.00	42.86*

^a^PELs = Protein expressed levels (Medium).

^b^% Protein identified = (Protein identified in each group / Total samples in each group)*100.

*Up-regulation.

In the categorization according to the biological process, most of the proteins were related to cellular (21.2%), metabolic (21.2%) processes, biological regulation (18.2%), signaling (12.1%), response to stimulus (12.1%), interspecies interaction between organisms (3.0%), developmental processes (3.0%), and immune system processes (3.0%), while 6.1% were unclassified. Moreover, the molecular function indicated that most proteins were involved with the catalytic activity and binding (44%) followed by transcription regulator (33.3%), and molecular function regulator (7.7%), while 7.7% were unclassified. Lastly, the cellular component category included proteins involved with the cellular anatomical entity (45.5%) and protein-containing complex (18.2%), while 36.4% were unclassified.

## Discussion

Proteomics has evolved as a useful tool for studying protein expression, protein interaction, protein modification, and discovery of new target molecules which provide information about the pathways and can deepen our insights of disease biology, host response, and host-pathogen interaction. An earlier study showed the importance of multiple malaria parasite infections. Multiple infections resulted in a higher proportion of severe anemia and pulmonary complications than those with mono-infection from *P*. *falciparum* [55]. In the natural environment, it is common for multiple infections to occur in wild macaques, but this assessment is not yet widely used. This led our research to focus on the comparison between non, mono and multiple-malaria parasite infection profiles in wild stump-tailed macaques residing in natural habitat. Our research proposed a method for generating and selecting protein subsets through statistical filtration methods, and both though unsupervised and supervised machine learning. Our study demonstrated that 440 DDEPs could be used as the most optimal set of protein features given their highest accuracy score. The enrichment of protein features in DDEPs was mapped by the KEGG pathway and we identified 142 important protein subsets which were analyzed by supervised machine learning. In terms of the host response to the malarial parasites, 64 proteins were involved with the environmental information processing and organismal systems pathways, while nine potential reporter proteins were involved with the interaction networks.

During the development of hepatocytes and erythrocytes, the *Plasmodium* parasite alters the host cell membrane and induces the formation of various membranous compartments such as parasitophorous vacuole membrane (PVM), parasitophorous vacuole (PV), and tubovesicular network (TVN) [56,57]. Previous studies have reported that the *Plasmodium* parasite can alter the host protein, lipid and fatty acid composition for their survival and proliferation within the host cell [58,59]. Other studies have suggested that lipids can play an important role in the synthesis of a membrane and are a source of energy for the cell. The *Plasmodium* parasite can regluate the AMP-activated protein kinase enzyme (AMPK) in the host hepatocytes [60]. Previously, it has been shown that AMPK is involved in the regulation of kinase activity to activate the AMP (AMPK) to hepatic lipid metabolism homeostasis during an infectious disease [61]. Previous studies have also indicated that some pathogens can stimulate AMPK activity due to an increased energy requirement for the host cell against pathogens such as *Leishmania* [57] and Simian vacuolating virus 40 [58]. In contrast, another experiment reported that murine malaria parasite infection in mice decreases the AMPK activity and make them vulnerable to a successful infection. Additionally, the activation of AMPK reduces the intracellular growth and replication of the *Plasmodium* parasite [62]. SREBF1 is a family of transcription factors that play an important role in the regulation of lipid biosynthesis and adipogenesis by regulating the gene controlling cellular lipid homeostasis [63,64]. In our proteomic data, SREBF1 was up-regulated in the infected macaques. Previous studies have concluded that the activation of AMPK suppresses the proteolytic activation of SREBF1, further reducing the expression of lipogenetic genes, reducing the triglyceride (TG) content in the hepatocytes [65,66]. The results of the present study suggest that SREBF1 was up-regulated in infected macaques, which may indicate that the *Plasmodium* parasite regulated the lipids and fatty acids of the host cell for survival and replication through the suppression of AMPK activity and upregulation of SREBF1. However, such a claim needs further study to elucidate this mechanism further.

We also detected an upregulation of RAF1 and NMPT in the infected macaques. RAF1 belongs to the RAF protein kinase family which participates in the Ras-RAF-MEK-ERk signaling pathway (MAPK signaling pathway) [67,68]. This activation stimulates a broad range of cellular signaling pathways, resulting in the regulation of a variety of cellular functions including cell proliferation, differentiation, apoptosis, autophagy, and neuronal differentiation [69,70]. A previous study had indicated that RAF1 promotes lymphatic metastasis of hypopharyngeal carcinoma by regulating the activity of LAGE1 gene [71]. Other experiments reported the activation of Ras-RAF-MEK-ERk stimulating the replication of Hepatitis C virus by downregulating the interferon-JAK-STAT pathway [70], while the Hepatitis B virus can regulate the RAF1 expression in HepG2.2.15 cell inducing hepatocellular carcinoma [67]. Moreover, the recent study indicated that multiple gene mutations of the Ras-MAPK signaling pathway can cause the Noonan syndrome (NS) which results in multiorgan disorder including bleeding and coagulation [72]. It is most commonly caused by a deficiency of factor XI followed by a reduced activity of factors VIII, XII, von Willebrand factor (vWF), thrombocytopenia, and platelet functional defect [72,73]. Our results from the protein interaction network indicate that RAF1 interacts with proteins responsible for clotting and blood coagulation. It has been demonstrated that cases of severe malaria can cause severe anemia and the development of disseminated intravascular coagulation (DIC) due to alteration of blood coagulation system, such as decreased plasma antithrombin levels, elevated levels of plasminogen activator (PAI)-1, reduced levels of vWF cleaving protease, ADAM metallopeptidase with thrombospondin type 1 motif 13 (ADAMTS13) and thrombocytopaenia, with poor chances of survival [74–76]. In addition, MEK1/2 was found to be an interesting host antimalarial drug target as it is involved with the regulation of meiosis, mitosis, and post-mitotic functions in different cells, several stimuli, including growth factors, cytokines, and infection. The regulation activity of MEK1/2 erythrocytes in the signaling pathway during the studied malarial infection has been shown to reduce soreness and multiplication of malarial parasites, as well as increase in the expression of anti-inflammatory cytokine and Th2 responses [77]. Here, we identified that RAF1 was up-regulated in the two infected groups. This protein is upstream of MEK1/2, making RAF1 a protein of potential interest for further study to be used as a target for host antimalarial drug. The NAMPT protein mainly controls the inflammatory and immune modulation [78] and catalyzes a key reaction in the intracellular Nicotinamide adenine dinucleotide (NAD) synthesis and acts as a damage-associated molecular model that activates the inflammatory response of Toll-like receptor 4 (TLR4) [79], and was identified in the two infected groups. Previous studies have shown that the concentration of NAMPT increases consistently in acutely infected samples, but for low-grade inflammation, NAMPT expression was not detected [80]. In addition, NAMPT is more likely to be secreted into the serum in presence of an acute infection, and this is consistent with the elevated production of white blood cells, making NAMPT one of the marker proteins that can be used to identify inflammatory conditions [81].

Type I interferons (IFN-Is) are an important host-produced basic cytokines responsible for mounting defense against several pathogens such as bacteria, fungi, and protozoa, including the malarial parasite [82,83]. The host response can recognize pathogen-associated molecular patterns (PAMPs) and danger-associated molecular patterns (DAMPs) using host-pathogen recognition receptors (PRRs), leading to the activation of various cytokines to inhibit the parasite [84]. PAMPS are small molecular forms of foreign substances that originate outside the cell, and include DNA, RNA, proteins, peptidoglycans, etc [85]. These molecules are recognized by PRRs, for example, Toll-like receptors (TLRs), cyclic GMP-AMP synthase (cGAS), nucleotide-binding oligomerization domain (NOD)-like receptors (NLRs), etc [84]. In this study, we identified two proteins in the antimalarial immune system through the parasite’s DNA and RNA. IRAK1 has also been identified as being uniquely up-regulated in the mono-infected group [86]. It has been reported to implicate the function of endosomes toll-like receptor 9 (TRL9), which binds to the unmethylated CpG motifs of a malarial parasite’s DNA [87]. Toll/interleukin-1 receptor (TIR) domain containing adaptor protein (TIRAP) was stimulated and bound with the myeloid differentiation primary response 88 (MYD88) and IRAK1 [88,89]. These signals were applied via IRF3/IRF7 to IFN-IS to mount the host defense. However, it remains a puzzling question as to whether the degree of severity of malaria affects the TRL9 function, as in this study, IRAK1 was only expressed in the mono-infected group, while it was down-regulated in the multiple-infected group. Subsequently, we identified CARD10 as the only protein as being up-regulated in the multiple-infected group. This protein is associated with the RNA of a malarial parasite and is recognized by the melanoma differentiation-associated protein 5 (MDA5) and activated by caspase activation recruitment domains (CARD). Activated MDA5 sends the signal to IRF3/IRF7 and IFN-IS, similar to DNA sensing [90,91].

TGF-β activity is responsible for reducing inflammation in the presence of malarial parasitic infection [92]. Several studies have reported on the changes in TGF-β levels when infected with the malarial parasite. In some cases, high levels of TGF-β were found in patients with acute malaria, while the levels were lower in patients with severe malaria [93–95]. SMAD7 functions as a key inhibitor of the TGF-signaling cascade and is released from the nucleus into the cytoplasm when TGF attaches to the TGF- receptor and initiates a downstream signaling cascade. It either prevents Smad2/4 from being phosphorylated or causes TGF- receptor I and Smad2/4 to degrade [96]. Additionally, SMAD7 acts as a negative regulator of SMAD2 and SMAD4. Our results show that in multiple-infected cohorts, there was a tendency to have a reduced TGF expression, as was the case with patients suffering from severe malaria in other studies [93–95].

Our results show that only one protein was down-regulated in the mono- and multiple-infected groups. SMARCC2 is an important myeloid differentiation regulator that also regulates the expression of genes involved in neutrophil granule formation and granulocytopoiesis. In general, during a malarial infection, neutrophils are one of the first lines of defense, as they are produced in large numbers during an infection. However, it is possible that when a parasite enters the hemolysis stage, it can result in the lysis of the oxidized heme and damage the surrounding cells, resulting in a decrease in cellular levels [97,98]. We observed that RELB was up-regulated in the non-infected and mono-infected groups, which is involved with the development of human lymphocyte. The growth of B cells is halted in the absence of RELB, which leads to an inadequate production of immunoglobulins and specific antibodies. A distorted T cell is produced and T cell output is lowered as they mature in the thymus [99]. Our research indicates a down-regulation of RELB in the multiple-infected group. Lastly, AKT2 was slightly up-regulated in the non-infected and multiple-infected groups. The difference in the expression of AKT2 was very little among the groups. However, it has been reported that a higher expression of AKT2 in the mosquitoe host reduces the prevalence of malarial parasitic infection [100].

## Conclusions

The present study is the first of its kind to look at the effect of malarial parasitic infection on the protein expression in wild stump-tailed macaques. We describe the sera proteomic analysis of wild stump-tailed macaques under non, mono, and multiple infections. We hypothesized that multiple-infection transmits severe pathogenesis compared to a mono-infection. We were able to identify nine reporter proteins involved in host defense mechanism through the inhibition of lipid metabolism, increase in apoptosis, cytokines, type I Interferons, and pro-inflammation. This included the parasite’s response through induced hemolysis, reduced TGF pathway, disruption of myeloid development and myeloid differentiation. Moreover, these proteins showed different expression trends under the three infection conditions. It was found that multiple infections were more likely to be stimulated through proinflammatory cytokines by host defense and take more damage from parasites than mono-infection and non-infection. A better understanding of the fundamental processes involved in a malarial infection and host response can be critical for the development of diagnostic tools, drugs, and therapeutics for improving the health of affected humans.

## Supporting information

S1 Data (XLSX)

## References

[1] Tuteja R. Malaria—an overview. The FEBS Journal. 2007;274(18):4670–9. doi: 10.1111/j.1742-4658.2007.05997.x 17824953

[2] Pasini EM, Kocken CHM. Parasite-Host Interaction and Pathophysiology Studies of the Human Relapsing Malarias Plasmodium vivax and Plasmodium ovale Infections in Non-Human Primates. Frontiers in Cellular and Infection Microbiology. 2021;10. doi: 10.3389/fcimb.2020.614122 33680982 PMC7925837

[3] World Health Organization. World Malaria Report 2021.

[4] van de Straat B, Sebayang B, Grigg MJ, Staunton K, Garjito TA, Vythilingam I, et al. Zoonotic malaria transmission and land use change in Southeast Asia: what is known about the vectors. Malaria Journal. 2022;21(1):109. doi: 10.1186/s12936-022-04129-2 35361218 PMC8974233

[5] Ramasamy R. Zoonotic malaria—global overview and research and policy needs. Front Public Health. 2014;2:123. doi: 10.3389/fpubh.2014.00123 25184118 PMC4135302

[6] Sam J, Shamsusah NA, Ali AH, Hod R, Hassan MR, Agustar HK. Prevalence of simian malaria among macaques in Malaysia (2000–2021): A systematic review. PLOS Neglected Tropical Diseases. 2022;16(7):e0010527. doi: 10.1371/journal.pntd.0010527 35849568 PMC9292078

[7] Fornace KM, Brock PM, Abidin TR, Grignard L, Herman LS, Chua TH, et al. Environmental risk factors and exposure to the zoonotic malaria parasite *Plasmodium knowlesi* across northern Sabah, Malaysia: a population-based cross-sectional survey. The Lancet Planetary Health. 2019;3(4):e179–e86. 31029229 10.1016/S2542-5196(19)30045-2 PMC6484808

[8] Bykersma A. The New Zoonotic Malaria: Plasmodium cynomolgi. Trop Med Infect Dis. 2021;6:46. doi: 10.3390/tropicalmed6020046 33916448 PMC8167800

[9] Ramasamy R. Zoonotic Malaria–Global Overview and Research and Policy Needs. Frontiers in Public Health. 2014;2:123. doi: 10.3389/fpubh.2014.00123 25184118 PMC4135302

[10] Muehlenbein MP, Pacheco MA, Taylor JE, Prall SP, Ambu L, Nathan S, et al. Accelerated diversification of nonhuman primate malarias in Southeast Asia: adaptive radiation or geographic speciation? Mol Biol Evol. 2015;32(2):422–39. doi: 10.1093/molbev/msu310 25389206 PMC4298170

[11] Faust C, Dobson A. Primate malarias: Diversity, distribution and insights for zoonotic Plasmodium. One Health. 2015;1. doi: 10.1016/j.onehlt.2015.10.001 28616467 PMC5441356

[12] Das Gupta B, editor Transmission of P. inui to man. Proc Natl Inst Sci India; 1938.

[13] Lalremruata A, Magris M, Vivas-Martínez S, Koehler M, Esen M, Kempaiah P, et al. Natural infection of *Plasmodium brasilianum* in humans: Man and monkey share quartan malaria parasites in the Venezuelan Amazon. eBioMedicine. 2015;2(9):1186–92. 26501116 10.1016/j.ebiom.2015.07.033 PMC4588399

[14] Fungfuang W, Udom C, Tongthainan D, Kadir KA, Singh B. Malaria parasites in macaques in Thailand: stump-tailed macaques (Macaca arctoides) are new natural hosts for Plasmodium knowlesi, Plasmodium inui, Plasmodium coatneyi and Plasmodium fieldi. Malaria Journal. 2020;19(1):350. doi: 10.1186/s12936-020-03424-0 33004070 PMC7528273

[15] Lempang M, Kresno Dewayanti F, Syahrani L, Permana D, Malaka R, Asih P, et al. Primate malaria: An emerging challenge of zoonotic malaria in Indonesia. One Health. 2022;14:100389. doi: 10.1016/j.onehlt.2022.100389 35686151 PMC9171520

[16] Chin W, Contacos PG, Coatney GR, Kimball HR. A Naturally Acquired Quotidian-Type Malaria in Man Transferable to Monkeys. Science. 1965;149(3686):865-. 14332847 10.1126/science.149.3686.865

[17] Singh B, Kim Sung L, Matusop A, Radhakrishnan A, Shamsul SS, Cox-Singh J, et al. A large focus of naturally acquired Plasmodium knowlesi infections in human beings. Lancet. 2004;363(9414):1017–24. doi: 10.1016/S0140-6736(04)15836-4 15051281

[18] Jongwutiwes S, Putaporntip C, Iwasaki T, Sata T, Kanbara H. Naturally Acquired Plasmodium knowlesi Malaria in Human, Thailand. Emerging infectious diseases. 2005;10:2211–3. 10.3201/eid1012.040293 PMC3323387 15663864

[19] Ng OT, Ooi EE, Lee CC, Lee PJ, Ng LC, Pei SW, et al. Naturally acquired human Plasmodium knowlesi infection, Singapore. Emerg Infect Dis. 2008;14(5):814–6. doi: 10.3201/eid1405.070863 18439370 PMC2600232

[20] Luchavez J, Espino F, Curameng P, Espina R, Bell D, Chiodini P, et al. Human Infections with Plasmodium knowlesi, the Philippines. Emerging infectious diseases. 2008;14:811–3. doi: 10.3201/eid1405.071407 18439369 PMC2600254

[21] Khim N, Sovannaroth S, Kim S, Mueller T, Fleischmann E, Singh B, et al. Plasmodium knowlesi Infection in Humans, Cambodia, 2007–2010. Emerging infectious diseases. 2011;17:1900–2. doi: 10.3201/eid1710.110355 22000366 PMC3310675

[22] Nada-Raja T, Kadir KA, Divis PCS, Mohamad DSA, Matusop A, Singh B. Macaca fascicularis and Macaca nemestrina infected with zoonotic malaria parasites are widely distributed in Sarawak, Malaysian Borneo. Scientific Reports. 2022;12(1):10476. doi: 10.1038/s41598-022-14560-9 35729212 PMC9213397

[23] Müller M, Schlagenhauf P. Plasmodium knowlesi in travellers, update 2014. Int J Infect Dis. 2014;22:55–64. doi: 10.1016/j.ijid.2013.12.016 24631521

[24] Biernat B, Lass A, Pietkiewicz H, Szostakowska B, Kuna A, Nahorski W. Investigations on the occurrence of Plasmodium knowlesi in travellers returning from the endemic areas of simian malaria. International maritime health. 2015;66:168–72. doi: 10.5603/IMH.2015.0033 26394318

[25] Thailand Malaria Elimination Program. Overview of Malaria Patients in Thailand 2022 2022.

[26] Putaporntip C, Jongwutiwes S, Thongaree S, Seethamchai S, Grynberg P, Hughes A. Ecology of malaria parasites infecting Southeast Asian macaques: Evidence from cytochrome b sequences. Molecular ecology. 2010;19:3466–76. doi: 10.1111/j.1365-294X.2010.04756.x 20646216 PMC2921002

[27] Sai-ngam P, Pidtana K, Suida P, Poramathikul K, Lertsethtakarn P, Kuntawunginn W, et al. Case series of three malaria patients from Thailand infected with the simian parasite, Plasmodium cynomolgi. Malaria Journal. 2022;21(1):142. doi: 10.1186/s12936-022-04167-w 35524255 PMC9074209

[28] Ta Tang TH, Hisam S, Lanza M, Jiram A, Ismail N, Rubio J. First case of a naturally acquired human infection with Plasmodium cynomolgi. Malaria journal. 2014;13:68. doi: 10.1186/1475-2875-13-68 24564912 PMC3937822

[29] Imwong M, Madmanee W, Suwannasin K, Kunasol C, Peto TJ, Tripura R, et al. Asymptomatic Natural Human Infections With the Simian Malaria Parasites Plasmodium cynomolgi and Plasmodium knowlesi. The Journal of Infectious Diseases. 2018;219(5):695–702. 10.1093/infdis/jiy519 PMC6376906 30295822

[30] Grignard L, Shah S, Chua T, William T, Drakeley C, Fornace K. Natural Human Infections With Plasmodium cynomolgi and Other Malaria Species in an Elimination Setting in Sabah, Malaysia. The Journal of infectious diseases. 2019;220. 10.1093/infdis/jiz397 PMC6834065 31418017

[31] Nada Raja T, Hu T, Kadir K, Mohamad D, Rosli N, Wong L, et al. Naturally Acquired Human Plasmodium cynomolgi and P. knowlesi Infections, Malaysian Borneo. Emerging infectious diseases. 2020;26:1801–9. doi: 10.3201/eid2608.200343 32687020 PMC7392409

[32] Putaporntip C, Kuamsab N, Pattanawong U, Yanmanee S, Seethamchai S, Jongwutiwes S. Plasmodium cynomolgi Co-infections among Symptomatic Malaria Patients, Thailand. Emerging Infectious Diseases. 2021;27:590–3. doi: 10.3201/eid2702.191660 33496236 PMC7853550

[33] Hartmeyer G, Stensvold CR, Fabricius T, Marmolin E, Høgh S, Nielsen H, et al. Plasmodium cynomolgi as Cause of Malaria in Tourist to Southeast Asia, 2018. Emerging infectious diseases. 2019;25:1936–9. doi: 10.3201/eid2510.190448 31538931 PMC6759256

[34] Yap NJ, Hossain H, Nada-Raja T, Ngui R, Muslim A, Hoh BP, et al. Natural Human Infections with Plasmodium cynomolgi, P. inui, and 4 other Simian Malaria Parasites, Malaysia. Emerg Infect Dis. 2021;27(8):2187–91. doi: 10.3201/eid2708.204502 34287122 PMC8314832

[35] Liew JWK, Bukhari FDM, Jeyaprakasam NK, Phang WK, Vythilingam I, Lau YL. Natural Plasmodium inui Infections in Humans and Anopheles cracens Mosquito, Malaysia. Emerg Infect Dis. 2021;27(10):2700–3. doi: 10.3201/eid2710.210412 34545786 PMC8462313

[36] Coatney GR, Collins WE, Warren M, Contacos PG. The primate malarias. 1971.

[37] Zhang X, Kadir K, Quintanilla-Zariñan L, Villano J, Houghton P, Du H, et al. Distribution and prevalence of malaria parasites among long-tailed macaques (Macaca fascicularis) in regional populations across Southeast Asia. Malaria journal. 2016;15:450. doi: 10.1186/s12936-016-1494-0 27590474 PMC5010671

[38] Eyles D, Laing A, Warren M, Sandosham A, Wharton R. Malaria parasites of the Malayan leaf monkeys of the genus Presbytis. Med J Malaya. 1962;17:85–6.

[39] Putaporntip C, Jongwutiwes S, Thongaree S, Seethamchai S, Grynberg P, Hughes AL. Ecology of malaria parasites infecting Southeast Asian macaques: evidence from cytochrome b sequences. Molecular Ecology. 2010;19(16):3466–76. doi: 10.1111/j.1365-294X.2010.04756.x 20646216 PMC2921002

[40] Jeyaprakasam NK, Liew JWK, Low VL, Wan-Sulaiman W-Y, Vythilingam I. Plasmodium knowlesi infecting humans in Southeast Asia: What’s next? PLOS Neglected Tropical Diseases. 2021;14(12):e0008900. 10.1371/journal.pntd.0008900 PMC7774830 33382697

[41] Ray S, Patel SK, Venkatesh A, Chatterjee G, Ansari NN, Gogtay NJ, et al. Quantitative Proteomics Analysis of Plasmodium vivax Induced Alterations in Human Serum during the Acute and Convalescent Phases of Infection. Scientific Reports. 2017;7(1):4400. doi: 10.1038/s41598-017-04447-5 28667326 PMC5493610

[42] Chan KC, Lucas DA, Hise D, Schaefer CF, Xiao Z, Janini GM, et al. Analysis of the human serum proteome. Clinical Proteomics. 2004;1(2):101–225.

[43] Ray S, Reddy PJ, Jain R, Gollapalli K, Moiyadi A, Srivastava S. Proteomic technologies for the identification of disease biomarkers in serum: advances and challenges ahead. Proteomics. 2011;11(11):2139–61. doi: 10.1002/pmic.201000460 21548090

[44] Kumar V, Ray S, Aggarwal S, Biswas D, Jadhav M, Yadav R, et al. Multiplexed quantitative proteomics provides mechanistic cues for malaria severity and complexity. Communications Biology. 2020;3(1):683. doi: 10.1038/s42003-020-01384-4 33204009 PMC7672109

[45] Ray S, Kamath KS, Srivastava R, Raghu D, Gollapalli K, Jain R, et al. Serum proteome analysis of vivax malaria: An insight into the disease pathogenesis and host immune response. J Proteomics. 2012;75(10):3063–80. doi: 10.1016/j.jprot.2011.10.018 22086083

[46] Cox-Singh J, Mahayet S, Abdullah MS, Singh B. Increased sensitivity of malaria detection by nested polymerase chain reaction using simple sampling and DNA extraction. Int J Parasitol. 1997;27(12):1575–7. doi: 10.1016/s0020-7519(97)00147-1 9467744

[47] Singh B, Bobogare A, Cox-Singh J, Snounou G, Abdullah MS, Rahman HA. A genus- and species-specific nested polymerase chain reaction malaria detection assay for epidemiologic studies. Am J Trop Med Hyg. 1999;60(4):687–92. doi: 10.4269/ajtmh.1999.60.687 10348249

[48] Lee K-S, Divis PCS, Zakaria SK, Matusop A, Julin RA, Conway DJ, et al. Plasmodium knowlesi: Reservoir Hosts and Tracking the Emergence in Humans and Macaques. PLOS Pathogens. 2011;7(4):e1002015. doi: 10.1371/journal.ppat.1002015 21490952 PMC3072369

[49] Lowry OH, Rosebrough NJ, Farr AL, Randall RJ. Protein measurement with the Folin phenol reagent. J Biol Chem. 1951;193(1):265–75. 14907713

[50] Johansson C, Samskog J, Sundström L, Wadensten H, Björkesten L, Flensburg J. Differential expression analysis of Escherichia coli proteins using a novel software for relative quantitation of LC-MS/MS data. Proteomics. 2006;6(16):4475–85. doi: 10.1002/pmic.200500921 16858737

[51] Thorsell A, Portelius E, Blennow K, Westman-Brinkmalm A. Evaluation of sample fractionation using micro-scale liquid-phase isoelectric focusing on mass spectrometric identification and quantitation of proteins in a SILAC experiment. Rapid Commun Mass Spectrom. 2007;21(5):771–8. doi: 10.1002/rcm.2898 17279600

[52] Belyadi HHA. Machine learning guide for oil and gas using Python: a step-by-step breakdown with data, algorithms, codes, and applications. 2021.

[53] Bertin GI, Sabbagh A, Argy N, Salnot V, Ezinmegnon S, Agbota G, et al. Proteomic analysis of Plasmodium falciparum parasites from patients with cerebral and uncomplicated malaria. Sci Rep. 2016;6:26773. doi: 10.1038/srep26773 27245217 PMC4887788

[54] Xu S, Li Y, Zheng W. Bayesian Multinomial Naïve Bayes Classifier to Text Classification2017. 347–52 p.

[55] Kotepui M, Kotepui KU, De Jesus Milanez G, Masangkay FR. Plasmodium spp. mixed infection leading to severe malaria: a systematic review and meta-analysis. Scientific Reports. 2020;10(1):11068. doi: 10.1038/s41598-020-68082-3 32632180 PMC7338391

[56] Sherling ES, van Ooij C. Host cell remodeling by pathogens: the exomembrane system in Plasmodium-infected erythrocytes. FEMS Microbiol Rev. 2016;40(5):701–21. doi: 10.1093/femsre/fuw016 27587718 PMC5007283

[57] Goldberg DE, Zimmerberg J. Hardly Vacuous: The Parasitophorous Vacuolar Membrane of Malaria Parasites. Trends in Parasitology. 2020;36(2):138–46. doi: 10.1016/j.pt.2019.11.006 31866184 PMC6937376

[58] Hsiao LL, Howard RJ, Aikawa M, Taraschi TF. Modification of host cell membrane lipid composition by the intra-erythrocytic human malaria parasite Plasmodium falciparum. Biochem J. 1991;274 (Pt 1)(Pt 1):121–32. doi: 10.1042/bj2740121 2001227 PMC1149929

[59] Ressurreição M, van Ooij C. Lipid transport proteins in malaria, from Plasmodium parasites to their hosts. Biochim Biophys Acta Mol Cell Biol Lipids. 2021;1866(12):159047. doi: 10.1016/j.bbalip.2021.159047 34461309

[60] Alsultan M, Morriss JM, Contaifer D, Kumar NG, Wijesinghe DS. Host Lipid Response in Tropical Diseases. Current Treatment Options in Infectious Diseases. 2020:1–15.

[61] Li Y, Xu S, Mihaylova MM, Zheng B, Hou X, Jiang B, et al. AMPK Phosphorylates and Inhibits SREBP Activity to Attenuate Hepatic Steatosis and Atherosclerosis in Diet-Induced Insulin-Resistant Mice. Cell Metabolism. 2011;13(4):376–88. doi: 10.1016/j.cmet.2011.03.009 21459323 PMC3086578

[62] Ruivo Margarida TG, Vera Iset M, Sales-Dias J, Meireles P, Gural N, Bhatia Sangeeta N, et al. Host AMPK Is a Modulator of Plasmodium Liver Infection. Cell Reports. 2016;16(10):2539–45. doi: 10.1016/j.celrep.2016.08.001 27568570 PMC5014760

[63] Horton JD, Goldstein JL, Brown MS. SREBPs: activators of the complete program of cholesterol and fatty acid synthesis in the liver. J Clin Invest. 2002;109(9):1125–31. doi: 10.1172/JCI15593 11994399 PMC150968

[64] Bertolio R, Napoletano F, Mano M, Maurer-Stroh S, Fantuz M, Zannini A, et al. Sterol regulatory element binding protein 1 couples mechanical cues and lipid metabolism. Nature Communications. 2019;10(1):1326. doi: 10.1038/s41467-019-09152-7 30902980 PMC6430766

[65] Hardie DG, Ross FA, Hawley SA. AMPK: a nutrient and energy sensor that maintains energy homeostasis. Nature Reviews Molecular Cell Biology. 2012;13(4):251–62. doi: 10.1038/nrm3311 22436748 PMC5726489

[66] Han Y, Hu Z, Cui A, Liu Z, Ma F, Xue Y, et al. Post-translational regulation of lipogenesis via AMPK-dependent phosphorylation of insulin-induced gene. Nat Commun. 2019;10(1):623. doi: 10.1038/s41467-019-08585-4 30733434 PMC6367348

[67] Tian Y, Hu Y, Wang Z, Chen K, Zhang L, Wang L, et al. Hepatitis B virus regulates Raf1 expression in HepG2.2.15 cells by enhancing its promoter activity. Arch Virol. 2011;156(5):869–74. doi: 10.1007/s00705-010-0901-z 21207082

[68] Simanshu DK, Nissley DV, McCormick F. RAS Proteins and Their Regulators in Human Disease. Cell. 2017;170(1):17–33. doi: 10.1016/j.cell.2017.06.009 28666118 PMC5555610

[69] Pearson G, Robinson F, Beers Gibson T, Xu BE, Karandikar M, Berman K, et al. Mitogen-activated protein (MAP) kinase pathways: regulation and physiological functions. Endocr Rev. 2001;22(2):153–83. doi: 10.1210/edrv.22.2.0428 11294822

[70] Zhang Q, Gong R, Qu J, Zhou Y, Liu W, Chen M, et al. Activation of the Ras/Raf/MEK pathway facilitates hepatitis C virus replication via attenuation of the interferon-JAK-STAT pathway. J Virol. 2012;86(3):1544–54. doi: 10.1128/JVI.00688-11 22114332 PMC3264379

[71] Li Y, Pan M, Lu T, Yu D, Liu C, Wang Z, et al. RAF1 promotes lymphatic metastasis of hypopharyngeal carcinoma via regulating LAGE1: an experimental research. Journal of Translational Medicine. 2022;20(1):255. doi: 10.1186/s12967-022-03468-7 35668458 PMC9172115

[72] Lan J, Zeng T, Liu S, Lan J, Qian L. Noonan syndrome with RAF1 gene mutations in a newborn with cerebral haemorrhage. European Journal of Medical Research. 2022;27(1):146. doi: 10.1186/s40001-022-00772-2 35953836 PMC9367063

[73] Kharel Z, Katel A, Neupane A, Panday P, Aryal M. Factor XIII Deficiency Associated With Noonan Syndrome. Cureus. 2021;13(3):e14150. doi: 10.7759/cureus.14150 33927952 PMC8076579

[74] Löwenberg EC, Charunwatthana P, Cohen S, van den Born BJ, Meijers JC, Yunus EB, et al. Severe malaria is associated with a deficiency of von Willebrand factor cleaving protease, ADAMTS13. Thromb Haemost. 2010;103(1):181–7. doi: 10.1160/TH09-04-0223 20062916

[75] Angchaisuksiri P. Coagulopathy in malaria. Thromb Res. 2014;133(1):5–9. doi: 10.1016/j.thromres.2013.09.030 24099998

[76] Riedl J, Mordmüller B, Koder S, Pabinger I, Kremsner PG, Hoffman SL, et al. Alterations of blood coagulation in controlled human malaria infection. Malaria Journal. 2016;15(1):15. doi: 10.1186/s12936-015-1079-3 26743539 PMC4705755

[77] Wu X, Dayanand KK, Thylur RP, Norbury CC, Gowda DC. Small molecule-based inhibition of MEK1/2 proteins dampens inflammatory responses to malaria, reduces parasite load, and mitigates pathogenic outcomes. J Biol Chem. 2017;292(33):13615–34. doi: 10.1074/jbc.M116.770313 28679535 PMC5566520

[78] Montecucco F, Cea M, Cagnetta A, Damonte P, Nahimana A, Ballestrero A, et al. Nicotinamide phosphoribosyltransferase as a target in inflammation- related disorders. Curr Top Med Chem. 2013;13(23):2930–8. doi: 10.2174/15680266113136660208 24171767

[79] Naing C, Wong ST, Aung HH. Toll-like receptor 9 and 4 gene polymorphisms in susceptibility and severity of malaria: a meta-analysis of genetic association studies. Malaria Journal. 2021;20(1):302. doi: 10.1186/s12936-021-03836-6 34217314 PMC8255014

[80] Gesing J, Scheuermann K, Wagner I, Löffler D, Friebe D, Kiess W, et al. NAMPT serum levels are selectively elevated in acute infectious disease and in acute relapse of chronic inflammatory diseases in children. PLoS One. 2017;12:e0183027. doi: 10.1371/journal.pone.0183027 28837586 PMC5570332

[81] Gesing J, Scheuermann K, Wagner IV, Löffler D, Friebe D, Kiess W, et al. NAMPT serum levels are selectively elevated in acute infectious disease and in acute relapse of chronic inflammatory diseases in children. PLoS One. 2017;12(8):e0183027. doi: 10.1371/journal.pone.0183027 28837586 PMC5570332

[82] Angulo I, Fresno M. Cytokines in the pathogenesis of and protection against malaria. Clin Diagn Lab Immunol. 2002;9(6):1145–52. doi: 10.1128/cdli.9.6.1145-1152.2002 12414742 PMC130117

[83] Silva-Barrios S, Stäger S. Protozoan Parasites and Type I IFNs. Frontiers in Immunology. 2017;8. doi: 10.3389/fimmu.2017.00014 28154565 PMC5243830

[84] Takeuchi O, Akira S. Pattern recognition receptors and inflammation. Cell. 2010;140(6):805–20. doi: 10.1016/j.cell.2010.01.022 20303872

[85] Tang D, Kang R, Coyne CB, Zeh HJ, Lotze MT. PAMPs and DAMPs: signal 0s that spur autophagy and immunity. Immunol Rev. 2012;249(1):158–75. doi: 10.1111/j.1600-065X.2012.01146.x 22889221 PMC3662247

[86] Jain A, Kaczanowska S, Davila E. IL-1 Receptor-Associated Kinase Signaling and Its Role in Inflammation, Cancer Progression, and Therapy Resistance. Frontiers in Immunology. 2014;5. doi: 10.3389/fimmu.2014.00553 25452754 PMC4233944

[87] Pichyangkul S, Yongvanitchit K, Kum-arb U, Hemmi H, Akira S, Krieg AM, et al. Malaria blood stage parasites activate human plasmacytoid dendritic cells and murine dendritic cells through a Toll-like receptor 9-dependent pathway. J Immunol. 2004;172(8):4926–33. doi: 10.4049/jimmunol.172.8.4926 15067072

[88] Kawai T, Akira S. Toll-like receptor and RIG-I-like receptor signaling. Ann N Y Acad Sci. 2008;1143:1–20. doi: 10.1196/annals.1443.020 19076341

[89] Kawai T, Akira S. The role of pattern-recognition receptors in innate immunity: update on Toll-like receptors. Nature Immunology. 2010;11(5):373–84. doi: 10.1038/ni.1863 20404851

[90] Liehl P, Zuzarte-Luís V, Chan J, Zillinger T, Baptista F, Carapau D, et al. Host-cell sensors for Plasmodium activate innate immunity against liver-stage infection. Nat Med. 2014;20(1):47–53. doi: 10.1038/nm.3424 24362933 PMC4096771

[91] Wang L, Guo Y, Huang W-J, Ke XL, Poyet J-L, Manji G, et al. CARD10 Is a Novel Caspase Recruitment Domain/Membrane-associated Guanylate Kinase Family Member That Interacts with BCL10 and Activates NF-κB. The Journal of biological chemistry. 2001;276:21405–9. 11259443 10.1074/jbc.M102488200

[92] Barral A, Barral-Netto M, Yong EC, Brownell CE, Twardzik DR, Reed SG. Transforming growth factor beta as a virulence mechanism for Leishmania braziliensis. Proc Natl Acad Sci U S A. 1993;90(8):3442–6. doi: 10.1073/pnas.90.8.3442 7682701 PMC46316

[93] Halsey ES, Baldeviano GC, Edgel KA, Vilcarromero S, Sihuincha M, Lescano AG. Symptoms and Immune Markers in Plasmodium/Dengue Virus Co-infection Compared with Mono-infection with Either in Peru. PLOS Neglected Tropical Diseases. 2016;10(4):e0004646. doi: 10.1371/journal.pntd.0004646 27128316 PMC4851334

[94] Nyirenda TS, Molyneux ME, Kenefeck R, Walker LS, MacLennan CA, Heyderman RS, et al. T-Regulatory Cells and Inflammatory and Inhibitory Cytokines in Malawian Children Residing in an Area of High and an Area of Low Malaria Transmission During Acute Uncomplicated Malaria and in Convalescence. J Pediatric Infect Dis Soc. 2015;4(3):232–41. doi: 10.1093/jpids/piu140 26335932 PMC4554200

[95] Chaiyaroj SC, Rutta AS, Muenthaisong K, Watkins P, Na Ubol M, Looareesuwan S. Reduced levels of transforming growth factor-beta1, interleukin-12 and increased migration inhibitory factor are associated with severe malaria. Acta Trop. 2004;89(3):319–27. doi: 10.1016/j.actatropica.2003.10.010 14744558

[96] Hu Y, He J, He L, Xu B, Wang Q. Expression and function of Smad7 in autoimmune and inflammatory diseases. Journal of Molecular Medicine. 2021;99(9):1209–20. doi: 10.1007/s00109-021-02083-1 34059951 PMC8367892

[97] Mooney JP, Galloway LJ, Riley EM. Malaria, anemia, and invasive bacterial disease: A neutrophil problem? Journal of Leukocyte Biology. 2019;105(4):645–55. doi: 10.1002/JLB.3RI1018-400R 30570786 PMC6487965

[98] Babatunde KA, Adenuga OF. Neutrophils in malaria: A double-edged sword role. Frontiers in Immunology. 2022;13. doi: 10.3389/fimmu.2022.922377 35967409 PMC9367684

[99] Sharfe N, Merico D, Karanxha A, Macdonald C, Dadi H, Ngan B, et al. The effects of RelB deficiency on lymphocyte development and function. J Autoimmun. 2015;65:90–100. doi: 10.1016/j.jaut.2015.09.001 26385063

[100] Corby-Harris V, Drexler A, Watkins de Jong L, Antonova Y, Pakpour N, Ziegler R, et al. Activation of Akt Signaling Reduces the Prevalence and Intensity of Malaria Parasite Infection and Lifespan in Anopheles stephensi Mosquitoes. PLOS Pathogens. 2010;6(7):e1001003. doi: 10.1371/journal.ppat.1001003 20664791 PMC2904800

